# Co-infections with *Borrelia species*, *Anaplasma phagocytophilum* and *Babesia* spp. in patients with tick-borne encephalitis

**DOI:** 10.1007/s10096-014-2134-7

**Published:** 2014-05-22

**Authors:** A. Moniuszko, J. Dunaj, I. Święcicka, G. Zambrowski, J. Chmielewska-Badora, W. Żukiewicz-Sobczak, J. Zajkowska, P. Czupryna, M. Kondrusik, S. Grygorczuk, R. Swierzbinska, S. Pancewicz

**Affiliations:** 1 Department of Infectious Diseases and Neuroinfections, Medical University of Białystok, 14 Zurawia, 15-540 Bialystok, Poland; 2 Department of Microbiology, Faculty of Biology and Chemistry, University of Białystok, 20 B Swierkowa, 15-950 Bialystok, Poland; 3 Department of Allergology and Environmental Hazards, Institute of Rural Health, 2 Jaczewskiego, 20-090 Lublin, Poland

## Abstract

The purpose of this study was evaluation of the prevalence of co-infection with *Borrelia species*, *A. phagocytophilum* and *Babesia* spp. in patients with tick-borne encephalitis (TBE). At total of 110 patients with TBE were included in the study. Serological tests for tick-borne encephalitis virus (TBEV), PCR for *Borrelia species*, *Anaplasma phagocytophilum* and *Babesia* spp., blood smears for *A. phagocytophilum* and *Babesia* spp. and BLAST analysis for *Babesia* spp. were performed. Results showed a significant majority of patients co-infected with *Borrelia species* (30/110; 27 %), much less with *A. phagocytophilum* (12/110; 10.9 %) and with *Babesia* spp. (1/110; 0.9 %). The BLAST analysis of the *18S rDNA* sequence obtained with the *Babesia* spp. specific primers indicated that the patient was infected with *Babesia microti*. Triple co-infections (TBEV-*Borrelia species- A. phagocytophilum*) were observed in three (3/110; 2.7 %) patients. Conclusions were such that differential diagnosis in patients after the tick bite, presenting with acute symptoms, should include not only TBE and Lyme disease, but also other diseases transmitted by ticks. In patients with low parasitemia in suspicion of *Babesia* spp. infection PCR seems to be a more sensitive method than blood smear. Co-infection with various tick-borne pathogens must be always considered, especially in endemic regions.

## Introduction

It is known that certain tick species are able to transmit more than one pathogen, e.g. tick-borne encephalitis virus (TBEV), *Borrelia burgdorferi* sensu lato (*Borrelia species*), *Anaplasma phagocytophilum* (*A. phagocytophilum*), and *Babesia species* (*Babesia* spp.), which may be a reason for co-infections [1]. Co-infections are becoming a serious epidemiological and clinical problem, especially due to the fact that the methods of treatment of infectious diseases caused by various tick-borne pathogens differ from each other. Therefore, this fact is more frequently considered in clinical research, especially in the areas endemic for tick-borne diseases. We suppose that positive results of PCR tests, especially in patients with non-specific symptoms in an early phase of infection, when antibodies cannot be detected with the help of traditional methods, may be helpful in the diagnosis and introduction of treatment of zoonoses such as Lyme disease (LD), tick-borne encephalitis (TBE), anaplasmosis or babesiosis.

## Objective

The objective of our study was to evaluate the prevalence of infection with *Borrelia species*, *A. phagocytophilum* and *Babesia* spp. in patients with TBE hospitalized between July 2009 and October 2012 in The Department of Infectious Diseases and Neuroinfections of Medical University of Bialystok, Poland.

## Material and methods

### Material

A group of 110 patients with TBE (47 female and 63 male) was included in the study. None of patients was vaccinated against TBE and all of them had a history of tick bites. Diagnosis was made on the basis of clinical manifestation, cerebrospinal fluid (CSF) examination and the presence of serum and CSF specific antibodies. Anti/TBEV antibodies titer was measured with *SERION ELISA classic TBE Virus IgG*/*IgM* (Institut Virion/Serion GmbH, Germany). Patients presented symptoms as shown in Table 1. None of them was immunocompromised. Laboratory tests and cerebrospinal tests results, as well as levels of TBEV antibodies are also presented in Table 1.

**Table 1:** Laboratory and cerebrospinal tests results, levels of tick-borne encephalitis virus (TBEV) antibodies and symptoms in TBE patients

Symptoms and laboratory tests results	TBE patients, *N* = 110
Headache	103 (93 %)
Vertigo	23 (21 %)
Nausea	40 (36 %)
Vomits	32 (30 %)
Fever	96 (87 %)
Muscle pain	20 (18 %)
Joint pain	20 (18 %)
*Erythema migrans*	0
Meningeal signs presence	90 (81 %)
Anti/TBEV IgM serum (mean) IU/ml	14 (cut off −0.45) 98 % of patients were positive
Anti/TBEV IgG serum (mean) IU/ml	30 (cut off –0.27) 95 % of patients were positive
Anti/TBEV IgM CSF (mean) IU/ml	4 (cut off −0.45) 80 % of patients were positive
Anti/TBEV IgG CSF (mean) IU/ml	8.8 (cut off –0.27) 82 % of patients were positive
CRP (mean ± SD) mg/dl	11.4 ± 12.5
SD (mean ± SD) mm/h	27.6 ± 15.7
WBC (mean ± SD) (tys)	9.1 ± 3.4
RBC (mean ± SD) (mln)	4.4 ± 0.5
PLT (mean ± SD) (×100 tys)	203.4 ± 56.5
CSF cytosis (mean ± SD) cells/μl	111 ± 154.6
CSF protein concentration (mean ± SD) (mg/dl)	63.9 ± 20.7

Serum was collected to search for anti/TBEV and anti/*Borrelia burgdorferi* antibodies. Whole blood in EDTA was used for PCR for *Borrelia species*, *Babesia* spp. and *A. phagocytophilum*. Blood smears were performed to search for *Babesia* spp. and *A. phagocytophilum* circulating stages. Cerebrospinal fluid was collected to perform biochemical, immunological and PCR examination.

A control group (CG) consisted of 20 healthy blood donors, in whom PCR for *Borrelia species*, *Babesia* spp. and *A. phagocytophilum* and blood smears were performed.

The study was approved by the Bioethical Commission of the Medical University of Bialystok.

### DNA isolation

DNA isolation was performed with the 200 μl of fresh or kept in +4 °C whole blood according to *QIAamp DNA Mini Kit* (Qiagen, Germany). DNA extracts in 100 μl volume were received from doubled elution by the mini spin column.

### PCR amplifications

For detection of particular tick-borne pathogens:* Borrelia species*, *Babesia* spp., *A. phagocytophilum* specific conservative genes were used. Sequence for protozoan *Babesia*: F2 (5′ GAC ACA GGG AGG TAG TGA CAA G 3′) and R2 (5′- biotin CTA AGA ATT TCA CCT CTG ACA GT 3′) amplifying a fragment from V4 region of *18S rDNA* gene [2] were synthesized by Sigma-Aldrich (Germany) and performed with *Taq PCR Core Kit* (Qiagen, Germany). In reaction for *Babesia* spp., 5 μl of extracted DNA were added to a reaction mixture (total volume of 50 μl) containing 5 μl of buffer x 10 with 15 mM MgCl_2_ (Qiagen, Germany), 2 μl of 25 mM MgCl_2_, 1 μl 10 mM dNTPs, 1 μl 20 μM of each primer and 0.25 μl (5U/μl) Taq DNA polymerase (Qiagen, Germany). The experimental constructed amplification programme included initial denaturation at 94 °C for 3 min, 40 cycles (denaturation at 94 °C for 40s, annealing at 58 °C for 60s, extension at 72 °C for 60s) and final extention at 72 °C for 10 min [3–5].

Amplification of *A. phagocytophilum* genetic material was performed with the diagnostic kit *PCR Anaplasma* (Blirt-DNA Gdańsk, Poland) coding a fragment of *16S rDNA* gene encoding small ribosomal 16S RNA subunit. Analyses were conducted in accordance with the manufacturers instruction, in the period from 2009 to 2011 in single PCR and in 2012 in a nested type of PCR. In conventional, single course PCR in 2011, 1 μl of the template DNA isolate was added to 43.7 μl of the Master Mix with 5 μl of dNTPs and 0.3 μl of *Hypernova* polymerase for a final reaction mix volume of 50 μl. Amplification was performed in the following PCR program: initial denaturation at 94 °C for 5 min, 35 cycles (denaturation at 94 °C for 30 s, annealing at 56 °C for 30 s, extension at 72 °C for 3 s) and final extension at 72 °C for 2 min. Positive results were 227 bp long fragments of the *16S rDNA* gene. Nested PCR for *A. phagocytophilum* DNA detection was performed in two amplifications. In the first, PCR-OUT 2 μl of the template DNA isolates was added to 42 μl of the Master Mix with 5 μl of dNTPs and 1 μl of *Taq nova* polymerase for a final reaction mix volume of 50 μl. First amplification was performed in the following PCR program: initial denaturation at 95 °C for 2 min, 40 cycles (denaturation at 94 °C for 30 s, annealing at 55 °C for 30 s, extension at 72 °C for 60 s) and final extension at 72 °C for 5 min. In a second amplification, PCR-IN, despite DNA isolate to 42 μl of the Master Mix with 5 μl of dNTPs and 1 μl of *Taq nova*, 2 μl of PCR product from first reaction was added. The PCR-IN program follows as in PCR-OUT, but in 30 cycles. Presence of the *16S rDNA* gene fragments: 932 bp long in PCR-OUT and 546 bp long in PCR-IN attest to *A. phagocytophilum* infection. Lack of 932 bp long fragments in PCR-OUT does not exclude a positive result of test.

#### Borrelia species

Molecular detection was performed by using the *Borrelia burgdorferi* PCR kit (GeneProof, Czech Republic) for in vitro diagnostics. The kit is designed for professional use in specialized clinical and research laboratories. The kit is designed for the detection of *Borrelia burgdorferi* sensu lato sp. group on the principle of amplification of the specific DNA sequence of a 276 bp fragment of flagellin encoding gene by nested one tube PCR. The template DNA extract was added to 36 μl of the MasterMix for a final reaction mix volume of 40 μl. “Hot start” technology was used in the detection kit, minimizing risk of non-specific reactions and maximizing sensitivity of procedure. Eventual PCR inhibition was controlled by internal standard in the reaction mix. Addition of uracil-DNA-glycosylase (UDG) eliminated possible contamination during preparation of the reaction. Nested PCR was performed in compatibility to GeneProof instruction with our own modifications. The course of the amplification was prepared according to the following reaction program: UDG decontamination at 37 °C for 2 min, initial denaturation at 96 °C for 10 min, first amplification for 30 cycles (denaturation at 96 °C for 20 s, annealing at 68 °C for 20 s, extension at 72 °C for 40 s), second amplification for 45 cycles (denaturation at 96 °C for 20 s, annealing at at 54 °C for 20 s, extension at 72 °C for 30 s) and final extension at 72 °C for 2 min.

**Fig. 1:**
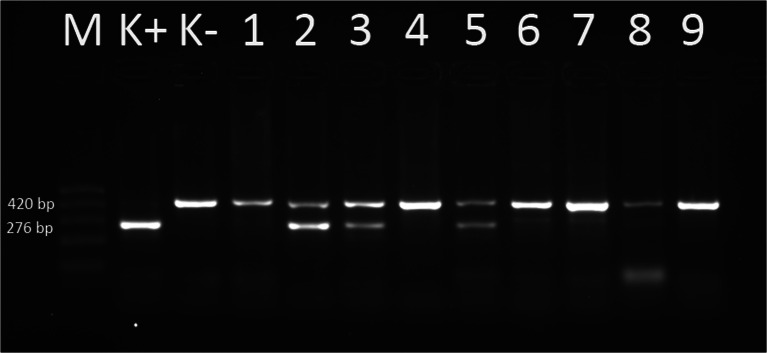
Electrophoretical detection of amplification of *Borrelia burgdorferi sl* PCR products on agarose gel. M 100–500 bp molecular weight marker; K+ positive control; K- negative control; 420 bp-internal standard; 276 bp positive *B. burgdorferi sl* fragments of *fla* gene; lines: 2,3,5 positive samples; lines: 1, 4, 6, 7, 8, 9 negative samples

**Fig. 2:**
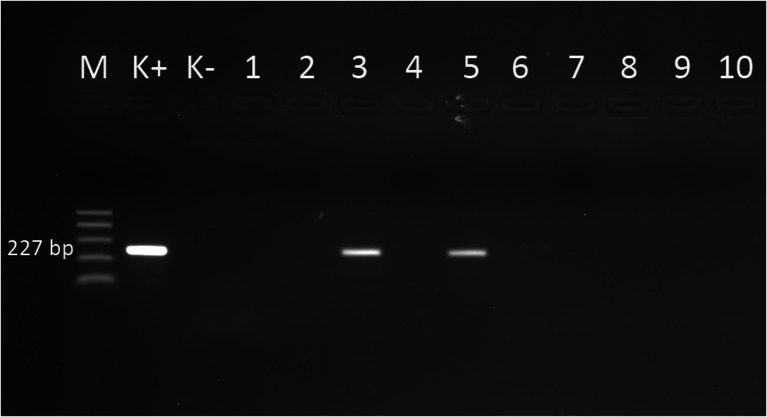
Electrophoretical detection of amplification of *Anaplasma phagocytophilum* PCR products on agarose gel. M 100–500 bp molecular weight marker; K+ positive control; K-negative control; 227 bp positive *Anaplasma phagocytophilum* fragments of *16S rDNA* gene; lines: 3, 5 positive samples, lines: 1, 2, 4, 6, 7, 8, 9, 10 negative samples

**Fig. 3:**
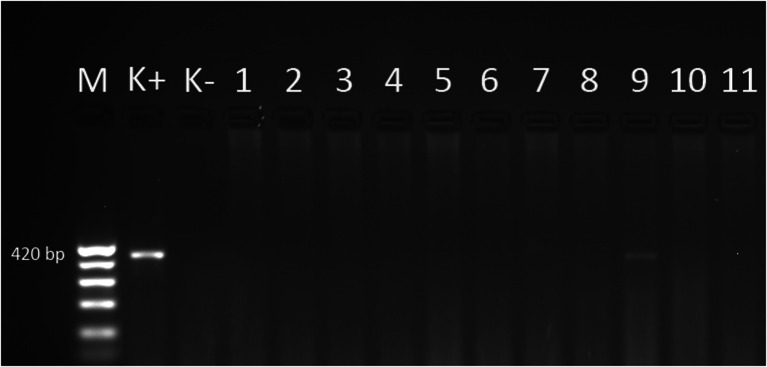
Electrophoretical detection of amplification of *Babesia species* PCR products on agarose gel. M 100–500 bp molecular weight marker; K+ positive control; K- negative control; 420 bp positive *Babesia species* fragments of *18S rDNA* gene; line: 9 positive samples; lines: 1, 2, 3, 4, 5, 6, 7, 8, 10, 11 negative samples

All amplifications were conducted on SensoQuest LabCycler (SensoQuest, Germany). Received PCR products were separated by electrophoresis in 2 % agarose gel (Sigma-Aldrich, Germany) stained with ethidium bromide (5 μg/1 ml; Syngen, USA). Electrophoresis conditions for *A. phagocytophilum* and *Babesia* spp. were 80 V by 60 min and for *Borrelia species* 80 V by 80 min. The results obtained were viewed under UV light and visualized by Gel Logic System 100 camera (Kodak, Imaging System, Inc., USA) (Figs. 1, 2, 3).

The amplification product for *Babesia* spp. showed 94 % homology to sequences received in Gen Bank NCBI in positions KC 581934.1, KC 470049.1, AY 789075.1 and AB 071177.1, which indicated *Babesia microti* presence.

For *A. phagocytophilum* the specificity and selectivity were determined by bioinformatic method using the NCBI database. Lack of specificity of tick DNA template kit was confirmed experimentally. Results of analysis showed that the system selectively detects only *A. phagocytophilum* DNA. In addition, the integrated package does not show homology to DNA templates of other prokaryotes or eukaryotes.

As positive controls we used DNA extracted from a deer spleen infected with *Babesia* spp.; in *Borrelia species* and in *A. phagocytophilum* cases a positive control was included in the particular kits. In a negative control redistilled water replaced DNA isolates. The size of amplification products for *fla* gene of *Borrelia species* was 276 base pairs (bp), for *Babesia* spp. *18S rDNA* approximately 420 bp and for *A. phagocytophilum* *16S rDNA* 227 bp in the 2009–2011 period and 546 bp in 2012.

### Nucleotide sequencing of the Babesia 18 rDNA amplicon

Amplification products of the *Babesia* spp. 18S rDNA were purified using the QiaAmp PCR purification kit (Qiagen). Sequencing reactions of both strands were performed using Big Dye Terminator cycle sequencing kit (Applied Biosystems) with the F2 and R2 primers under the same conditions as above. Cycle sequencing reactions were purified using the ExTerminator Kit (A&A Biotechnology, Gdynia, Poland) and sequenced with the ABI3500 automated sequencer (Applied Biosystems, Foster City, USA). The obtained sequences were compared with sequences collected in the NCBI database (http://www.ncbi.nlm.nih.gov).

### Immunoserology diagnostic

The detection of TBE virus infection was performed with *SERION ELISA classic TBE Virus IgG*/*IgM* (Institut Virion/Serion GmbH, Germany) diagnostic kit twice in patients with meningitis or encephalitis, namely, at the time of admission to the hospital and 2 weeks later. Anti/TBEV antibody level dynamics were observed. A level of viral specific IgM and IgG antibodies was marked according to manufacturer’s recommendations.

Anti/*B. burgdorferi* IgM and IgG antibodies in enzyme-linked immunosorbent assay (ELISA; Borrelia recombinant IgG and IgM High Sensitivity, Biomedica, Austria) and intrathecal production of anti/*B. burgdorferi* antibodies (EcoLine test, Virotech Classic Kits, Germany) in cases with suspicion of neuroborreliosis were performed.

### Blood smears

Obligatory, from full blood collected on EDTA, blood smears stained with May-Grunwald and Giemsa (MGG) were performed twice. Piroplasm forms in erythrocytes were searched for *Babesia* spp. infection and morulae in neutrophiles for *A. phagocytophilum* infection.

### Statistical analysis

Statistical analysis to compare patients with only TBE infection and co-infections was performed using Statistica 10. Groups were compared by Mann–Whitney and Pearson’s chi-squared tests. *P* value <0.05 was considered statistically significant.

## Results

In our study, conducted in the northeastern part of Poland on a group of 110 patients with TBE, a significant majority was co-infected with *Borrelia *species (30/110; 27 %), much less with *A. phagocytophilum* (12/110; 10.9 %) and with *Babesia* spp. (1/110; 0.9 %). The BLAST analysis of the 18S rDNA sequence obtained with the *Babesia* spp. specific primers indicated that the patient was infected with *Babesia microti*. One patient was diagnosed as neuroborreliosis, based on anti/*B. burgdorferi* IgM and IgG serum antibodies and intrathecal production of anti/*B. burgdorferi* antibodies in CSF.

Triple co-infections (TBEV-*Borrelia species- A. phagocytophilum*) were observed in three (3/110; 2.7 %) patients.

In blood smear we observed no piroplasm forms in erythrocytes typical of *Babesia* spp. infection or morulae in neutrophiles typical of *A. phagocytophilum* infection.

Analysis of patients infected only with TBEV and patients co-infected with other pathogens showed no significant difference in the presented symptoms, which indicates overlapping of symptoms in cases of TBEV infection. However, there were significant differences in the laboratory parameter values such as the erythrocyte sedimentation rate (ESR) (*p* = 0.028) and alanine aminotransferase activity (*p* = 0.006) (Tables 2 and 3).

**Table 2:** Comparison of symptoms between patients only with tick-borne encephalitis (TBE) and patients with co-infections

Symptoms	TBEV (*N* = 70)		TBEV + *B. burgdorferi* sl TBEV + *A. phagocytophilum* TBEV + *Babesia* spp. (*N* = 40)		*p*
	*n*	%	*n*	%	
Headaches	66	94.3 %	37	92.5 %	0.71
Vertigo	12	17.2 %	11	27.5 %	0.19
Nausea	23	33 %	17	42.5 %	0.31
Vomiting	22	31.4 %	10	25 %	0.47
Muscle pain	11	15.7 %	11	27.5 %	0.13
Joint pain	11	15.7 %	9	22.5 %	0.37
Fever	62	88.6 %	32	80 %	0.21
Meningeal signs presence	53	75.7 %	24	60 %	0.08
Neck stiffness	44	63 %	29	72.5 %	0.3

*TBEV* tick-borne encephalitis virus

**Table 3:** Comparison of laboratory test results between patients only with tick-borne encephalitis (TBE) and patients with co-infections

Laboratory parameters	TBEV (*N* = 70)			TBEV + *B. burgdorferi* sl TBEV + *A. phagocytophilum* TBEV + *Babesia* spp. (*N* = 40)			*p*
	Mean	SD	Median	Mean	SD	Median	
ESR (mm/h)	34	21	30	45	19	46	0.028
CRP (mg/l)	9.9	10.8	7.5	14.4	15.2	8.4	0.16
RBC (mln/μl)	4.45	0.54	4.4	4.48	0.44	4.6	0.47
Hemoglobin (g/dl)	13.5	1.45	13.5	13.6	1.1	13.7	0.75
Hematocrit (%)	40.1	8.3	39.9	39.4	3.4	39.5	0.9
WBC (tys/μl)	9.03	3.2	9.02	9.13	4	9.3	0.98
PLT (tys/μl)	208	59	208	194	51	194	0.3
ALT (U/l)	34	37	22	21	25	13	0.006
AST (U/l)	28	23	21	20	8	17	0.14
LDH (U/l)	315	514	199	236	72	238	0.28
Creatinine (mg/dl)	0.76	0.15	0.74	0.77	0.17	0.78	0.62
Glucose (mg/dl)	99	16	99	103	31	95	0.71
Fibrynogen (mg/dl)	464	196	415	455	144	474	0.88
Bilirubin (mg/dl)	1.25	1.6	0.62	0.67	0.27	0.57	0.92

*TBEV* tick-borne encephalitis virus

## Discussion

In Poland, similarly to other European countries, tick-borne diseases are an increasing epidemiological and clinical problem. Annual incidence of LD has also been increasing systematically. The number of registered cases in 2005 and 2009 was 4.406 (incidence 11.5/100.000 inhabitants) and 10,333 cases (incidence 27.1/100.000 inhabitants), respectively. During a period of 5 years a 2-fold increase in LD incidence has been observed. The regions considered to be endemic are the Podlaskie and Warminsko-Mazurskie regions (northeastern Poland) with annual 5-fold higher incidence in comparison to the whole country. In 2012, a total of 9.159 cases were reported (incidence 24/100.000 inhabitants) [6].

TBE incidence has also been increasing for years. The number of registered cases in 2005 and 2009 was 174 (incidence 0.46/100.000 inhabitants) and 344 (incidence 0.9/100.000 inhabitants), respectively. In 2012, 188 cases were reported (incidence 0.49/100.000 inhabitants). Similarly to LD most cases have been observed in the Podlaskie and Warminsko-Mazurskie regions [6].

At the moment the exact number of cases suffering from anaplasmosis and babesiosis is unknown. At the same time, it is known that from 1956, only in Europe, more than 50 cases of human babesiosis have been confirmed [7–9]. Human Granulocytic Ehrlichiosis (HGE, anaplasmosis) has been diagnosed from 1994 in the United States, from 1996 in Europe and from 2001 in Poland [7]. Despite that, only a few cases have been described. For example, due to the research of Welc-Falęciak et al., in a group of 30 tick-exposed people from southeastern Poland only one case of *Babesia spp*. (1/30; 3.3 %) and one of *A. phagocytophilum* (1/30; 3.3 %) infection were confirmed with PCR [8].

It is known that more than one pathogen may co-exist in one vector. According to various European sources, co-infection of *I. ricinus* ticks with different pathogens appears to be quite common [5, 10–13]. In Poland, the prevalence of co-infected *I. ricinus* ticks with at least two pathogens varies from 0.12–8.30 % and depends on the area of tick sampling [1, 14–16].

Human co-infection with various pathogens may be the result of a single tick bite by the tick infected with more than one pathogen or the result of multiple bites by ticks infected with one pathogen. Both situations may result in a co-infection, often difficult to diagnose and differentiate [17]. Meer-Scheerer et al. described a case of *B. microti* and *Borrelia species* co-infection [18]. Krause et al. observed that in 1156 serosurvey subjects, 97 (8.4 %) were seroreactive against LD spirochete antigen, of whom 14 (14 %) also were seroreactive against babesial antigen [19]. Varis et al. described patients simultaneously infected with TBEV and *B. burgdorferi* spirochete as a result of a single tick bite [20]. Cimperman et al. also identified patients who had ELISA serum IgM and IgG antibodies of TBEV and a positive PCR result for TBEV in cerebrospinal fluid as well as *Borrelia species* isolated from cerebrospinal fluid [21]. Hermanowska-Szpakowicz et al. observed patients with LD (8/96; 8.3 %) and patients with TBEV (4/96; 4.1 %) coinfected only with *A. phagocytophilum*, but not with *Babesia microti* [7].

In our study conducted in northeastern Poland we observed a rate of co-infections of 2.7 %: *B. burgdorferi*/*A. phagocytophilum*/TBEV. However, we observed a quite high rate of co-infection with TBEV and *Borrelia species* (27 % of patients with TBEV were positive for *Borrelia species*), which was not observed in other studies. It may be explained by pre-selections of patients, who were admitted to hospital due to suspected meningitis. We also noticed a patient with *Babesia microti* infection, which was asymptomatic or with mild course. This observation may suggest significantly higher importance of *Babesia* infection than it has been considered previously.

Various methods may be used to diagnose infections caused by tick-borne agents. Among these are serological examinations (TBE, LD, babesiosis, anaplasmosis), PCR (LD, TBE, babesiosis, anaplasmosis) and blood smear (babesiosis, anaplasmosis) [22]. Each of these methods might be used separately and it is not necessary to have positive results of all of them simultaneously. The ideal situation would be to obtain the same results from all available methods; however, none of them has 100 % specificity and sensitivity. Blood smear, which in some cases may be negative, is the best example of problems in the diagnostic process. In general, the analysis of blood smears is a fairly subjective process. The need to discriminate the subtleties of babesial/anaplasmal morphology and possible low parasitemias may result in inaccurate diagnoses, which might require further analysis.

In cases of anaplasmosis suspicion 25–75 % of patients have morulae in peripheral blood smear examinations, with the highest sensitivity during the first week of infection [23]. Only one-third of patients with babesiosis have piroplasm forms in erythrocytes in microscopic study [21]. Additionally, Aktas et al. observed in sheep blood samples that only in four out of 98 piroplasms *B. ovis* forms were present in thin blood smears, whereas in PCR 21 samples were positive [24]. If there is a strong suspicion of anaplasmosis or babesiosis and parasitemia is low, detection of parasites may by difficult on a thin blood smear, and molecular techniques are recommended [22].

From the clinical point of view the influence of co-infections on the course of disease and treatment administration is the most important issue. Logina et al. analyzed 51 patients with double infection—TBEV and *Borrelia species*—and concluded that the clinical occurrence of both LD and TBE varies after exposure to tick bite, and the neurological manifestations of each disorder differ significantly, with appreciable overlap [25]. However, their study provided no proof that co-infection manifested with extraordinary symptoms due to unexpected interaction between these two pathogens. Nevertheless, they suggest that all the patients from endemic areas presenting with acute neurological symptoms after the tick bite should be investigated for both LD and TBE and simultaneous treatment of both conditions should be introduced as quickly as possible.

In another study of 687 patients with TBE 2 % were diagnosed with neuroborreliosis. They more frequently had pleocytosis of over 300 cells/mm³ and statistically higher concentration of protein (88.2 mg/dl vs 67.4 mg/dl). In the group without neuroborreliosis, symptoms like headaches, vertigo, nausea and vomiting were more frequent than neurological symptoms in comparison to the group with neuroborreliosis [26].

On the other hand, Alekseev et al. suggested that *Borrelia species* might suppress viral replication in ticks and in TBE-susceptible individuals, hence it is still a matter of controversy [27]. In our study no differences in clinical picture between patients with only TBE infection and co-infection with other tick-borne pathogens were stated.

Grab et al. suggested that *A. phagocytophilum* co-infection contributes to the severity, dissemination and possible sequelae of LD. They showed that co-infection enhanced reductions in transendothelial electrical resistance and enhanced or synergistically increased production of metalloproteinases, cytokines and chemokines, which are known to affect vascular permeability and inflammatory responses [28].

Co-infection with *Babesia* and *Borrelia species* may influence the clinical course, especially in non-immunocompetent patients, and might be difficult to diagnose due to the fact that both diseases cause nonspecific symptoms, such as fever, fatigue, and flu-like illness. Patients with an inadequate response to appropriate therapy for proven or suspected LD following a tick bite should be examined for infections with other tick-borne agents, including *Babesia* species [28]. It is also worth remembering during travel to endemic areas for tick borne diseases, as babesiosis is starting to appear as a travel-related disease [29].

We assume that administration of antibiotic therapy in cases suspected of co-infection should be introduced as soon as possible and should be verified after the advanced laboratory test results, such as molecular biology methods (e.g. PCR). We also should be aware that number of pathogens transmitted on humans by ticks is constantly increasing (e.g. *Fransicella tularensis*, *Bartonella* spp., spotted fever rickettsiae group and many others) and further studies are necessary [30].

## Conclusions

1. Nonspecific symptoms after tick bite may results from presence of other than TBEV pathogens, not diagnosed routinely.
2. In patients with low parasitemia in suspicion of *Babesia* spp. PCR seems to be a more sensitive method than blood smear.
3. Co-infection with various tick-borne pathogens must always be considered, especially in regions endemic for these diseases.
